# Bovine tuberculosis in eastern Ethiopia: prevalence, risk factors and its public health importance

**DOI:** 10.1186/s12879-018-3628-1

**Published:** 2019-01-10

**Authors:** Jelalu Kemal, Berhanu Sibhat, Aklilu Abraham, Yitagele Terefe, Ketema Tafess Tulu, Kiros Welay, Nejib Getahun

**Affiliations:** 1 Haramaya University College of Veterinary Medicine, P. O. Box 138, Dire Dawa, Ethiopia; 2 Haramaya University College of Health and Medical Science, Dire Dawa, Ethiopia; 3 Department of Health Technology and Informatics, The Hong Kong Polytechnic University, Hong Kong SAR, China; 4 Department of Medical Laboratory, College of Health Sciences, Arsi University, Asella, Ethiopia; 5 Haramaya University School of Animal and Range Science, P. O. Box 138, Dire Dawa, Ethiopia

**Keywords:** Bovine tuberculosis, CIDT test, Eastern Ethiopia, Public health, Risk factors.

## Abstract

**Background:**

Bovine tuberculosis is among the primary zoonotic disease caused by *Mycobacterium bovis* which has significant impact on the health of livestock and human. It has been significantly a cause for great economic loss in animal production.

**Methodology:**

A cross-sectional study was conducted from December 2014 to June 2016 on 315 cattle in selected areas of eastern Ethiopia, aiming to estimate the occurrence of bovine tuberculosis using comparative intradermal tuberculin skin test and assess cattle owners’ awareness on its public health implication. Random sampling method was applied in order to select animals from farm/household and associated risk factors were recorded before purified protein derivative (PPD) injection. Forty three farm/household owners of tuberculin tested animals were interviewed using pre-tested structured questionnaires.

**Results:**

The overall prevalence of bovine tuberculosis was 20.3% (*n* = 64) in dairy cattle at recommended cut off > 4 mm. From a total of 43 farms/households tested, 22 were positive; each farm exhibited at least one tuberculin positive reactor animal with a total herd level prevalence of 51.2%. The prevalence of bovine tuberculosis in individual animal level was significantly different (χ2 = 45.2; *P*-value = 0.000) in different sites with a higher prevalence (50%) in Dire Dawa. Farming system, herd size and other risk factors were significantly (*p* < 0.05) associated with bovine tuberculosis occurrence. Of the total interviewed farm owners, only 33% had the knowledge of or had heard about bovine tuberculosis and 23% respondents were aware of the zoonotic importance of the disease. More than 50% of the interviewees had shown their preference of raw milk consumption. Out of the total interviewed households, 3 (7%) farm workers had TB cases that had direct contact with the animals.

**Conclusion:**

The study showed bovine tuberculosis is highly prevalent. Associated risk factors contributed to the prevalence of the disease in cattle and its transmission. Moreover, the majority of cattle owners lack awareness about the disease and its public health significance. Awareness rising about the disease, its transmission and zoonotic implication is of great importance for reduction and control measures. Evidence of tuberculosis patient farm attendants calls also for further detail investigation.

## Introduction

A close interaction between animals and humans primarily contributes to the transmission of infectious zoonotic diseases between them [1]. Bovine tuberculosis is a common zoonotic disease caused by *Mycobacterium bovis* which affects a wide range of animals and humans [2, 3]. Cattle based tuberculosis has become a significant infectious disease which spread between species. Bovine tuberculosis is widely distributed around the world with significant economic impact on the livestock production sector [4–6]. It has been recorded as the most recurrent cause of zoonotic tuberculosis in human [7].

In developed countries, the occurrence of human tuberculosis due to *M. bovis* has meaningfully declined because of mandatory pasteurization of milk together with tuberculin skin testing of cattle followed by culling/slaughtering the infected cattle [8]. However, in developing countries particularly in Africa, it represents a potential health threat to both humans and animals. This is mainly because closely 82% of the human and 85% of cattle population live in regions where BTB is highly prevalent [9]. Studies showed that BTB is still common in these developing countries where routine milk pasteurization is not practiced, and an estimated 10–15% of human tuberculosis incidences are because of *M. bovis* [10, 11].

Investigation of *M. bovis* both in animal and human population still leftover low in such underdeveloped regions particularly in Africa despite the disease remains a possible health risk. Several factors such as social turbulence, political uncertainty, and related cost of testing programme, wars leading to people and animal displacement, and shortage of skilled expertise in the field considerably influenced investigation of the disease on its public health implication [12]. Among the African countries, BTB is highly prevalent disease in Ethiopia in cattle population. Some survey results performed using intradermal tuberculin skin test in Ethiopia shows that the occurrence of BTB varies from 0.8–78% in free range rural farming systems that predominantly keep Zebu cattle and intensive systems that keep exotic and cross breed cattle respectively [12–14].

Few studies have also indicated as the disease is zoonotic it transmitted from animal to humans and vice versa [15, 16]. Many other studies have revealed that there are many potential associated risk factors that are conducive to the spreading and persistence of BTB in developing countries. Some of these includes eating habits, educational status, demography, living style, socio-economic status, culture, existence of other immunosuppressive diseases, and sharing the same house with animals [4, 16, 17]. Unpasteurized raw milk is preferably consumed than boiled milk in Ethiopian community mainly because of its accessibility, convenience, good taste and lower price. The public health threat of BTB is mainly associated with consumption of unpasteurized dairy products having *M. bovis*. Close contact between animals and humans is also considered as a potential risk factor for the disease [18, 19].

In Ethiopia the distribution of BTB is not well-known in livestock population, and most studied works have been focused mainly around the central part of the country mainly in and around Addis Ababa. The current prestige of the disease should have to be investigated in all regions of the country where such study has not yet been done in order to go aboard the national control program of BTB in the future. The objectives of this study were to estimate the prevalence of bovine tuberculosis in selected areas of eastern Ethiopia, namely; Harar, Dire Dawa, and Jigjiga with comparative intradermal tuberculin test (CIDT), identify associated risk factors and assess dairy cattle owners’ awareness on its public health.

## Materials and methods

### Study area description

This study was conducted in selected areas of eastern Ethiopia viz.; Harar, Dire Dawa and Jigjiga, Ethiopia.

**Fig. 1:**
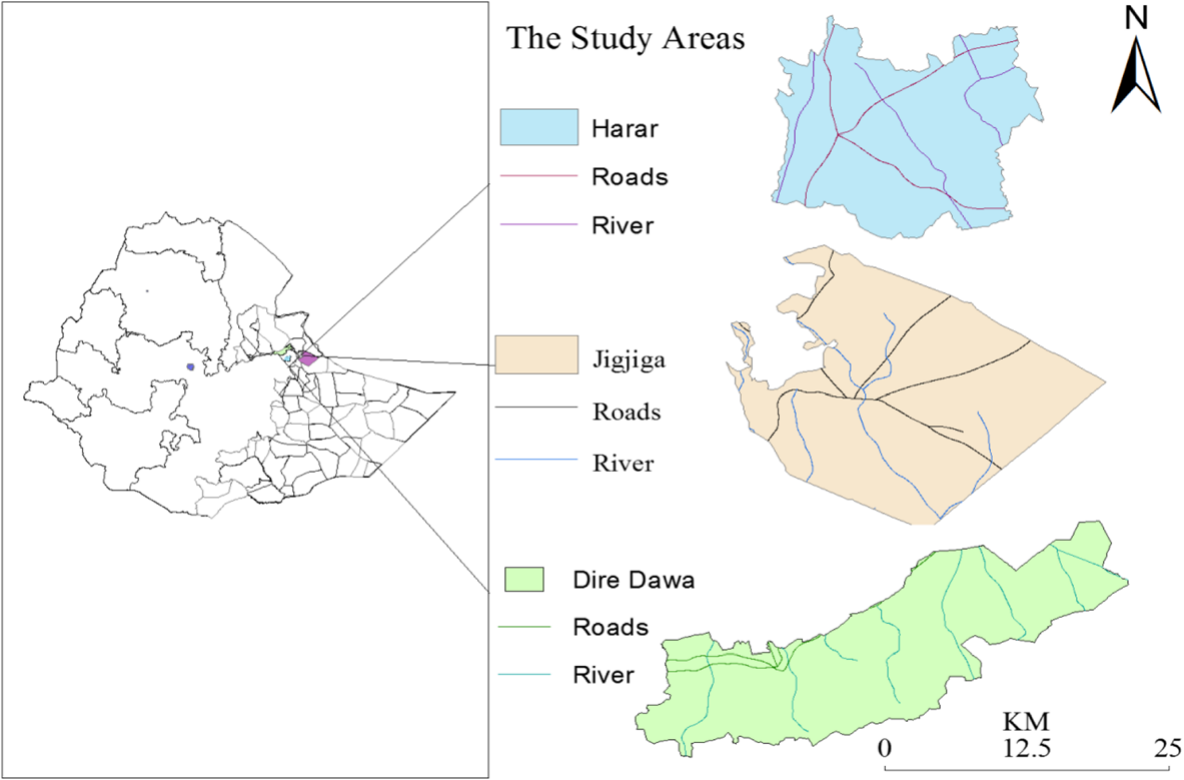
Illustrative representation of study areas (Harar, Jigjiga, and Dire Dawa)

Harar is the administrative capital of Harari Region which is one of the nine National Regional States of Ethiopia. It has a total population of 183,344, of whom 99,321 (54.17%) are urban inhabitants. The region is divided in to 9 districts and 36 lower administrative units (Kebeles). Harar is located 526 km far from Addis Ababa in East direction at a latitude of 8°500′-9°15’N and longitude of 9°36′N 41°52′ East and situated at an altitude of 1850 m above sea level (m.a.s.l) (Fig. 1). The annual rainfall of the area is between 834 and 1300 mm. This area experiences a binominal rainfall pattern with a long rainy season from June to September and short rainy season from March to April while the annual temperature ranges from 21 to 26 °C [20]. The CSA [20] estimated that farmers in Harari had a total 44,199 head of cattle, 36,320 goats, 4130 sheep, 6320 equine, 1400 camel, 31,430 poultry of all species and 670 bee hives.

Dire Dawa is one of the second largest urban settlements in Ethiopia next to Addis Ababa, the capital of Ethiopia. It is located at about 515 km to the east of Addis Ababa. The area is found between 9°27′ and 9°49’ N latitude and 41°38′ and 42°19′ E longitude (Fig. 1). The total area of the administration is 128,802 ha. It shares common boundaries with Somali Regional State in the west, north, and east and with the Oromia Regional State in the south. Administratively, it is divided into 9 urban Kebeles (lower administrative unit) and 32 peasant associations. The rural area occupies 98.7% of the total land area. The altitude of Dire Dawa Administration (DDA) ranges from 960 m.a.s.l in the northeast to 2450 m.a.s.l. in the southwest. The mean annual rainfall of the area varies from 550 mm in the lowland northern part to 850 mm in the southern mountains with average 640 mm. The monthly mean minimum and maximum temperature ranges from 14.5 °C to 34.6 °C respectively. Out of the rural population about 4% are pastoralists engaged in livestock production as the only livelihood activity. The total livestock population in DDA is estimated to be 219,323. Goats comprise the highest proportion (54.2%) followed by sheep (21.1%) and cattle (18.4%) [20].

Jigjiga is the capital of Ethiopian Somali Region which is the most eastern among the nine Regional States of the country. The Region borders with Dire Dawa Administration, Oromia and Afar Regions to the west, republics of Djibouti and Somalia to the north, east and south and Kenya to the south-west. Jigjiga is the one among the nine Administrative Zones of the Region. It has an urban inhabitant’s number of 621,210 (14%) of the population. It is located 615 km from Addis Ababa in East direction with an altitude ranging from 1660 to 1850 m.a.s.l. It is geographically located at 8^o^ 44’N longitude and 40^o^ 22′E latitude with mean minimum and maximum temperatures of around 17 °C to 30 °C, respectively (Fig. 1). According to National Meteorological Service Agency reports, the mean annual rain fall is 660 mm and is bimodal in its distribution. The livestock population of the district is estimated to be 62,156 cattle, 100,516 sheep, 142,048 goats, 10,172 equines, 17,185 poultry, 12,825 camels and 695 bee hives. The vegetation cover mainly includes dwarf and large shrubs and trees (such as *Boscia* and *Acacia* species) and highly populated cacti. Livestock herding is a prevalent profession of the rural population with free range pastoralist and agro­pastoralist management as the most dominant production systems. The majority (78.33%) of the farmers raise both crops and livestock, while 19.88% only grow crops and 1.79% raises only livestock [20].

### Study animals and sample size determination

Cattle reared in the study areas were considered as the study animals. Extensive, semi-intensive and intensive types of cattle production systems were practiced in the areas. Cattle production using improved breeds (Holstein-Friesians, Jersey and crossbred of Holstein-Friesians with Zebu) is a common practice in urban and some peri-urban areas. In semi-intensive farming system animals are allowed access to grazing typically in the morning and evening and supplied with silage, brewery by-products, hay and other concentrates when they are housed in well-constructed shades. In rural areas, mainly local breeds (Zebu) are found kept grazing on communal land under traditional animal farming system. The main purpose of the farms is to produce milk for household consumption and for commercial purposes. One institutional farm kept dairy cows to generate income and for provision of the community within and around the institute with milk for fair price and for teaching purposes. The sample size for tuberculin testing was calculated using the sampling formula described by Thrusfield [21] with the expected prevalence of 8.6% (based on true prevalence) [22] and absolute precision value of 5%. The minimum sample size required to estimate the prevalence of BTB was 121 cattle. However, considering the small sample size, the design effect (to account for clustering of cases in a herd), and resources available to carry out the study, the sample size was re-calculated using statistical calculator in Epi Info™ 7 by choosing the population survey option [23]. Assuming design effect of 2.4, the sample size required from the selected 43 clusters (households/farms) would be 301 cattle above the age of six months with an average of 7 cattle sampled per farm. In the current study 315 animals were sampled more than 2.5 times the sample size for simple random sampling.

### Study design and sampling method

A cross-sectional study was conducted from December 2014 to June 2016 on 315 cattle in eastern Ethiopia to estimate the prevalence of bovine tuberculosis and assess cattle owners’ awareness on its association with public health. List of households/farms owning dairy cattle were obtained from the offices of rural development agents of the Livestock and Fishery Department. The lists of households/farms were used as sampling frames. The households/farms were considered as primary unit and the individual animals as secondary units. Herds of livestock in the households/farms of the study areas were grouped in to three (small, medium and large herd size) based on herd size. From each study area, both herd and individual animals were selected randomly. Two animals from small herd size to eight animals from large herd size above 6 months of age were randomly selected per household/farm. Lottery sampling technique was employed to select the animals. No samples lose occurred during the study period. One month pre and post-partum cattle were excluded during the study.

Associated risk factors considered for data collection at animal and herd levels were recorded before purified protein derivatives (PPD) were injection. Impermanent unique identification numbers were given for each tested animal. Body condition score (BCS) of the animals was determined according to Nicholson and Butterworth [24] as poor, medium or good. Poor body condition score were considered for extremely lean cattle with projecting dorsal spines pointed to the touch and individual noticeable transverse processes. Cattle with usually visible ribs having little fat cover and barely visible dorsal spines was expressed as a medium body condition score. A good body condition score was articulated for the animals when fat cover is easily observed in critical areas and the transverse processes were not visible or felt. The management condition (sanitation status) of the studied farms was categorized as poor, medium (satisfactory), or good as described by Ameni et al. [22] based on housing condition such as odor, neatness, waste drainage, nature and cleanness of the floor and animals, light source, ventilation, presence of confinement), feeding practice (concentrate and hay), possession of an exercise yard, and contact with other nearby herds and provision with clean water. Physiological conditions of cattle were categorized as pregnant or non-pregnant and lactating or as non-lactating. The age of studied cattle was categorised into 1–5, 6–9 and > 9 years. Herd size was characterized into three groups. These were: small herds (1–5 cattle), medium herds (6–10 cattle), and large herds (> 10 cattle). Majority of the investigated farms (70%) were farms that hold more than 10 cattle that were categorized under large scale herds.

### Comparative intradermal tuberculin skin test

Both avian and bovine mycobacterium PPDs were used to perform the CIDT test. Intradermal injections were made 12–15 cm apart from each other in the skin of the middle neck of study cattle [3]. After shaving the site, the thickness of the shaved skin fold was measured to the nearest millimeters using digital calipers and measurements were recorded before PPD injection. Aliquots of 3000 IU/0.1 ml bovine PPD (Veterinary Laboratories Agency, Addlestone, Surrey, United Kingdom) plus tuberculin comprising 2500 IU/0.1 ml avian PPD (Veterinary Laboratories Agency, Addlestone, Surrey, United Kingdom) were inoculated intradermal at the lower and upper injection sites respectively. The inoculated skin site was palpated for occurrence of small swelling in order to check accuracy. Then, the thicknesses of the previously PPD injected skin folds at both sites were measured and recorded after 72 h of inoculation. The result interpretation of both bovine and avian positive reactors was gained based on the skin fold differences of bovine PPD (B) and avian PPD (A) using the formula given by OIE [3]. This skin thickness difference (B-A) results were interpreted based on OIE [3] recommended cut off > 4 mm in which skin fold thickness of above 4 mm was considered as positive reactor while skin thickness less than this record was considered as negative reactor.

### Questionnaire survey

A total of 43 farm/herd owners were interviewed using pre-tested structured questionnaires to know the knowledge and awareness level of the owner of the study area. They were interviewed regarding BTB and its transmission associated with feeding habits of dairy products and other related factors such as sharing the same house with cattle and consumption habit of raw milk of the owners or herders. In addition, information on TB status of the herders and farm workers was gathered using a designed questionnaire format during the period. Each farm owner or attendant was interviewed in the local languages (using Amharic, Afaan Oromoo and Somaligna) depending on the preference of the respondent at the time the CIDT was done. The interview was made voluntarily from all cattle owners and attendants that participated in the study.

### Data analysis

The overall data obtained from tuberculin skin testing were logged in the format prepared for the purpose and then were entered in to Microsoft Excel spreadsheet. STATA version 11.0 was used for analysis of the data. Prevalence of individual animal was demarcated as the number of positive reactor animals per 100 tested animals. Prevalence in the farm level was considered as the number of herds with at least one-reactor animal per 43 tested herds. Univariable logistic analysis was used to compute the properties of different potential risk factors. Goodman and Kruskal’s Gamma statistic was used to check multicollinearity in a cross-tabulation for all risk factors. All the non-collinear predictors (gamma values between − 0.6 to + 0.6) with generous *p*-values of equal or less than to 0.2 were considered in the improvement of the multivariable logistic regression model, and using backward elimination technique, the final model was developed. This was done based on Wald’s test and likelihood ratio test statistics (*p* < 0.05). By constructing two-product terms the interactions between predictors were tested for the significant main effect variables, forcing them into the model and examining changes in OR and p-values of the main effects. Confounding effect were also checked using the changes in the proportion of OR. Then, a covariate was considered to be a confounder and comprised in the model if its inclusion altered the OR of the estimated risk at least by 20%. The Hosmer and Lemeshow modeling method was used to assess the final model for goodness-of-fit and the receiver operating curve (ROC) for reliability Dohoo et al. [25].

## Results

### Individual animal and herd level BTB prevalence

The overall prevalence of BTB with comparative intra-dermal tuberculin test was 20.3% (95% confidence interval = 16.0–25.2) (*n* = 64) in dairy cattle in the areas. From a total of 43 farms/households tested, 22 were positive; each farm exhibited at least one tuberculin positive reactor animal with a total herd level prevalence of 51.2% (Table 1).

**Table 1:** Prevalence of bovine tuberculosis in animal and herd level

Study site	Animal level prevalence			χ2	*P*-value	Herd level prevalence			χ2	*P*-value
	No. Tested	Positive	Prevalence (%)			No. Tested	Positive	Prevalence (%)		
Harar	224	25	11.2	45.2	0.000	35	16	45.7	2.36	0.306
Dire Dawa	58	29	50.0			5	4	80.0		
Jigjiga	33	10	30.3			3	2	66.7		
Total	315	64	20.3			43	22	51.2		

### Animal level risk factors associated with bovine tuberculosis prevalence

The prevalence of bovine tuberculin positivity at individual animal level was 20.3% at cut-off > 4 mm. Different potential animal level risk factors for the occurrence and transmission of BTB were indicated. Age, lactation, pregnancy, site of the study, farming type, farming system, herd size and management condition were significantly (*p* < 0.05) associated with BTB prevalence in the areas (Table 2). Collinear variables ‘pregnancy’ and ‘farming system’ were not included in the final multivariable regression model building. Accordingly only study area, lactation status and management conditions were significantly associated (*p* < 0.05) with individual animal BTB skin reaction (Table 3). The Hosmer and Lemeshow method for goodness-of-fit and the ROC results are presented in Table 3.

**Table 2:** Evaluation of the association of animal level risk factors with prevalence of bovine tuberculin positivity

Risk factor*	Categories	No. of animals		OR (95% CI)	*P*-value
		Examined	Positive (%)		
Site	Harar	224	25 (11.2)	Ref.	
	Dire Dawa	58	29 (50.0)	7.96 (4.107–15.427)	0.000
	Jigjiga	33	10 (30.3)	3.46 (1.477–8.104)	0.004
Age	1–5 years	85	17 (20.0)	Ref.	
	6–9 years	172	42 (24.4)	1.29 (0.685–2.439)	0.429
	> 9 years	58	5 (8.6)	0.37 (0.130–1.089)	0.072
BCS	Poor	67	15 (22.4)	Ref.	
	Medium	147	29 (19.7)	0.85 (0.421–1.721)	0.655
	Good	101	20 (19.8)	0.85 (0.402–1.820)	0.686
Lactation^a^	Non-lactating	115	11 (9.5)	Ref.	
	Lactating	200	53 (26.5)	0.29 (0.146–0.588)	0.001
Pregnancy^a^	Non pregnant	276	51 (18.5)	Ref.	
	Pregnant	39	13 (33.3)	2.22 (1.062–4.587)	0.034
Breed^b^	Local	63	11 (17.5)	Ref.	
	Cross breed	15	1 (6.7)	0.30 (0.032–2.794)	0.289
	Exotic	237	52 (21.9)	0.71 (0.195–2.596)	0.607
Herd size^c^	1–5	46	5 (10.9)	Ref.	
	6–10	48	4(8.3)	0.74 (0.187–2.968)	0.677
	> 10	221	55(24.9)	2.71 (1.022–7.218)	0.045
Farming system^b,c^	Extensive	51	7 (13.7)	Ref.	
	Intensive	229	50 (21.8)	3.69 (0.820–16.625)	0.089
	Semi intensive	35	7 (20.0)	1.84 (0.418–8.120)	0.419
Management condition	Good	83	1 (1.2)	Ref.	
	Poor	63	14 (22.2)	3.66 (1.31–1.83)	0.000
	Medium	169	49 (29.0)	1.51 (0.51–1.51)	

*Variables with similar superscripts are collinear

**Table 3:** Multivariable logistic regression analysis of risk factors associated with individual animal bovine tuberculosis status

Variables	Categories	Odds ratio (95% CI)	*P*-value
Site	Harar	Ref.	
	Dire Dawa	3.9 (1.9–7.8)	< 0.001
	Jijiga	1.6 (0.6–4.1)	0.305
Lactation status	Non-lactating	Ref.	
	Lactating	2.5 (1.2–5.3)	0.018
Management conditions	Poor	Ref.	
	Medium	1.2 (0.5–2.4)	0.710
	Good	0.06 (0.01–0.5)	0.008

Hosmer-Lemeshow χ^2^ = 5.46, *p* = 0.49; area under ROC = 0.7907

### Assessment of cattle owners’ awareness on the public health importance of BTB

Of the total 43 farm/household owners and/or members of these households/farms interviewed for assessment of awareness on the zoonotic effect of the disease, 14 (32.55%) reported that they had the knowledge of or had heard about BTB and 10 (23.25%) respondents were aware of the zoonotic importance of BTB. Out of the total interviewed households, 3 (6.97%) farm workers/attendants had TB cases that had direct contact with the animals and within two of the three households there had been both PPD-positive reactor cattle and human tuberculosis cases. Moreover, cattle owners were also interviewed regarding their raw milk drinking and raw meat eating and house sharing habits with their animals (Table 4).

**Table 4:** Summary of cattle owners’ knowledge about BTB and its transmission to humans

Question item	Number of respondents’	Number responded (%)
Know BTB can affect animal	43	14 (32.55)
Know BTB is zoonotic	43	10 (23.25)
Know raw milk is vehicle for TB	43	9 (20.93)
Consume raw milk	43	23 (53.48)
Know meat is vehicle for BTB	43	8 (18.60)
Consume raw meat	43	14 (32.55)
Share the same house with cattle	43	16 (37.20)
Know close contact with cattle can facilitate BTB transmission	43	5 (11.62)
Sick with TB	43	3 (6.97)

## Discussion

In the current study the overall prevalence of bovine tuberculin test positivity at individual animal and herd level was 20.3 and 51.2%, respectively. The herd level prevalence was found to be higher than the prevalence of individual animals in the study that could most probably be due the herd size of farms that can favour BTB transmission in exhaustive dairy farms in particular [22, 26]. The finding is relatively in line with Firdessa et al. [27] who found more than 30 and 58% individual animal and herd level BTB prevalence, respectively which was conducted in dairy cattle at central Ethiopia. Relative findings were reported by Ameni et al. [28] with individual animal prevalence of 24.3, 27.3 and 27.8% at Holleta, Ziway and Ambo dairy farms respectively. Shitaye et al. [26] found 18.7% animal level BTB prevalence that was conducted at Addis Ababa with a total of 2098 examined animals.

A higher animal level prevalence than the current study was reported by Ameni et al. [28] with BTB prevalence of 65.8 and 73.6% at Debre Zeit and Dessie, respectively. Comparative BTB prevalence was reported by Elias et al. [29] with 23.7% animal level and 43.4% herd level prevalence. Tsegaye et al. [30] found 34.1 and 53.6% individual animal and herd level BTB prevalence respectively. Higher overall individual animal prevalence of 46.8% and a herd prevalence of 91.7% were recorded in 12 dairy farms by the CIDT test [28]. These prevalence variations among different researchers finding could be due to geographical location, socioeconomic aspect of the areas, study methodology (study design and test procedure), production systems, herd size, breed type, production system and others.

Our finding was in contrary with the findings of Ameni and Erkihun [15] who reported 11 and 15% prevalence of BTB at animal and herd level, respectively in Adama town (Oromia state). Gebremedhin et al. [31] (northern Ethiopia), Tschopp et al. [32] (central Ethiopia) and Mohammed et al. [33] (northwest Ethiopia) reported 6.6, 6.8 and 7.1% overall animal level prevalence respectively. Low BTB prevalence was reported by different researchers conducted in different areas of the country. Gumi et al. [34] found the individual animal prevalence of 2.0% in cattle. Tschopp et al. [9] reported very low BTB prevalence with an individual animal level prevalence of 0.3%.

The multivariable logistic regression analysis of risk factors in this current study found that lactation status, study site, and management conditions of the farms were significantly associated with PPD reactivity in intradermal skin test (Table 3). Cattle from Dire Dawa Administration showed higher (nearly four times) BTB skin reaction than those in Harar and Jigjiga. This statistically significant infection variation between the three farms might probably be due to agro-ecological and climate features (heat) differences among the farms in which the temperature reaches up to 35 °C in Dire Dawa Administration. Moreover, cattle of *Bos taurus* (European breeds) become stressed when they kept in more hot and overcrowded environment which can be expressed as possible explanations for high prevalence of BTB in the area. Scott et al. [35] suggested high heat limitation had an effect in favor of *Mycobacterium* transmission among animals. In addition to climatic feature differences in between the study areas, the dairy housing system of the sites also differs that cattle kept under indoor system and house with poor ventilation may facilitate transmission of *the pathogen* between cattle.

According to the multivariable logistic regression analysis, lactating cows (OR = 2.5) were more than two and half times more reactive to bovine tuberculin positivity as compared to non-lactating cows. In comparison with our finding, Elias et al. [29] reported that among physiological status group, lactating animals had the highest prevalence of BTB. Inangolet et al. [36] reported lactating female animals had higher BTB prevalence than non-lactating animals. Similarly, Kazwala et al. [37] found significantly more (14.6%) PPD reacted lactating cattle in the skin test than did non-lactating cows (12.0%). This significant difference could possibly be due to greater production stress experienced by dairy cows and also gathering of cows during milking which promotes the risk of transmission of the disease [38]. In contrast to the current study, Romha et al. [39], Zeru et al. [19] and Dejene et al. [40] found insignificant association between lactating and non-lactating cattle with the prevalence of BTB in their study.

Tuberculin positivity result also revealed a statistically significant association with herd management conditions indicating that poor managerial inputs increase the risk of BTB. Farms that are managed poorly were 3.66 times more likely to be test positive for BTB than farms having good management status. Previous study [41] had reported higher BTB infection in farms under poor management conditions. O’Reilly and Daborn [42] noted that higher probability of susceptibility to BTB infection to genetically improved cattle breed kept under poor housing than good and well managed farm. This finding is also supported by other previous studies that had reported higher BTB infections prevalence in farms under poor management situations [24, 30, 36, 43, 44]. This can be concluded as the occurrence of BTB could be improved by implementing sanitary measures that improve hygiene situations on farms.

Assessment on the level of awareness of cattle owners about BTB showed that 32.55% of the respondents know cattle can be infected by tuberculosis, and 23.25% recognized that BTB is zoonotic. High number of respondents had therefore, no detailed and accurate knowledge about tuberculosis and its zoonotic importance. Ameni et al. [14] and Radostits et al. [41] have indicated that lack of understanding regarding the zoonotic effect of BTB, food consumption behavior and poor sanitary measures are among the potential risk factors of BTB to public health. Similarly, around 53.48% of the respondents were consuming raw milk indicating most of milk consumers do prefer raw milk than treated milk due to the taste, availability and lower price of raw milk [18]. Only 21 % (20.93%) and (18.60%) of the interviewed farm owners and/or farm attendants know milk and meat could be vehicles for zoonotic transmission of BTB, respectively.

The survey indicated 37.20% of the respondents shared the same house with their cattle. Transmission of the disease tuberculosis may be cyclical i.e. cow-to-man-to-cow [12], underlying the existence of risk of dissemination of the pathogen among human and cattle population. Humans attain the infection mainly by ingesting the causative agent in raw milk and its products as well as by inhaling the pathogen during close physical contact between the owner and his/her cattle, particularly at night time since in some cases they share shelters with their animals [45]. Our assessment of the knowledge of the society on BTB is in line with the findings of Mohammed et al. [33], Sisay et al. [46], Akililu et al. [47] and Fikre et al. [48].

At the same time, in relation with previous history of TB in farm workers, the questionnaire revealed 6.97% (3 out of 43) of the respondents had history of TB. One of the farm attendants who had TB (confirmed to be cured with long time medication after several clinical examinations at the time of the interview) explained to have close and unreserved contacts with dairy cattle in different farms for 5 years. The attendant listed symptoms of the disease as night sweating and superficial wounds mainly around neck and back. The patient reported to have suffered for several months with a misdiagnosis and wrong prescription (treatment) before being diagnosed as a TB patient. PPD reactor cattle were recorded in two of the farms where the mentioned patient served. This calls for further detailed investigation to determine the source of infection and direction of TB transmission.

## Conclusion

The present study showed that BTB is prevalent both in households and intensive dairy farms with individual animal prevalence of 20.3%. Herd level prevalence was 51.2%. BTB prevalence increased with increasing herd size and with farms having poor management condition..The questionnaire survey of this study revealed that the majority of cattle owners’ in the area lack awareness of BTB and its public health significance. As a result, large portion of the community had habit of drinking raw milk and sharing the same shelter with close contact implying the possible potential of acquiring BTB from positive animals. Thus, awareness should be created on BTB transmission and its public health significance to cattle owners and farm attendants for effective implementation of TB control measures. Evidence of tuberculosis patients among farm attendants could indicate transmission of the disease from animal to human or vice versa calling for further detailed investigation in order to point out and address the possible source and way of transmission of the disease.

## Abbreviations

BCS: Body condition score
BTB: Bovine Tuberculosis
CIDT: Comparative Intradermal Tuberculin Test
CSA: Central Statistical Agency
PPD: Purified Protein Derivative
